# Immunosuppression by monocytic myeloid-derived suppressor cells in patients with pancreatic ductal carcinoma is orchestrated by STAT3

**DOI:** 10.1186/s40425-019-0734-6

**Published:** 2019-09-18

**Authors:** Rosalinda Trovato, Alessandra Fiore, Sara Sartori, Stefania Canè, Rosalba Giugno, Luciano Cascione, Salvatore Paiella, Roberto Salvia, Francesco De Sanctis, Ornella Poffe, Cristina Anselmi, Francesca Hofer, Silvia Sartoris, Geny Piro, Carmine Carbone, Vincenzo Corbo, Rita Lawlor, Samantha Solito, Laura Pinton, Susanna Mandruzzato, Claudio Bassi, Aldo Scarpa, Vincenzo Bronte, Stefano Ugel

**Affiliations:** 1 University Hospital and Department of Medicine, Section of Immunology, University of Verona, Verona, Italy; 2 Present Address: Max Planck Institute of Biochemistry, Martinsried, Germany; 3 Department of Computer Science, University of Verona, Verona, Italy; 4 Institute of Oncology Research, Bellinzona, Switzerland; 5 General and Pancreatic Surgery, Pancreas Institute, University of Verona, Verona, Italy; 6 Medical Oncology, Fondazione Policlinico Universitario Agostino Gemelli, IRCCS, Rome, Italy; 7 Faculty of Medicine and Surgery, Università Cattolica del Sacro Cuore, Rome, Italy; 8 Department of Department of Diagnostic and Public Health, University of Verona, Verona, Italy; 9 ARC-Net Centre for Applied Research on Cancer, University and Hospital Trust of Verona, Verona, Italy; 10 Department of Surgery, Oncology and Gastroenterology, Section of Oncology and Immunology, University of Padova, Padova, Italy; 11 Present Address: Centro Piattaforme Tecnologiche (CPT), University of Verona, Verona, Italy; 12 Istituto Oncologico Veneto IOV-IRCCS, Padova, Italy

**Keywords:** Myeloid-derived suppressor cells (MDSC), Pancreatic ductal adenocarcinoma (PDAC), Innate immunity, Tumor-associated immunosuppression, Tumor progression

## Abstract

**Background:**

Pancreatic ductal adenocarcinoma (PDAC) is a highly devastating disease with an overall 5-year survival rate of less than 8%. New evidence indicates that PDAC cells release pro-inflammatory metabolites that induce a marked alteration of normal hematopoiesis, favoring the expansion and accumulation of myeloid-derived suppressor cells (MDSCs). We report here that PDAC patients show increased levels of both circulating and tumor-infiltrating MDSC-like cells.

**Methods:**

The frequency of MDSC subsets in the peripheral blood was determined by flow cytometry in three independent cohorts of PDAC patients (total analyzed patients, *n* = 117). Frequency of circulating MDSCs was correlated with overall survival of PDAC patients. We also analyzed the frequency of tumor-infiltrating MDSC and the immune landscape in fresh biopsies. Purified myeloid cell subsets were tested in vitro for their T-cell suppressive capacity.

**Results:**

Correlation with clinical data revealed that MDSC frequency was significantly associated with a shorter patients’ overall survival and metastatic disease. However, the immunosuppressive activity of purified MDSCs was detectable only in some patients and mainly limited to the monocytic subset. A transcriptome analysis of the immunosuppressive M-MDSCs highlighted a distinct gene signature in which STAT3 was crucial for monocyte re-programming. Suppressive M-MDSCs can be characterized as circulating STAT3/arginase1-expressing CD14^+^ cells.

**Conclusion:**

MDSC analysis aids in defining the immune landscape of PDAC patients for a more appropriate diagnosis, stratification and treatment.

## Background

Over the last thirty years, pancreatic ductal adenocarcinoma (PDAC) worldwide incidence has increased significantly and PDAC ranks the fourth leading cause of cancer death with a 5-year survival time of less than 8% [1]. Despite many new treatments, including immune modulation, pancreatic cancer remains highly resistant to therapy [2, 3]. The presence of the highest degree of desmoplasia among all solid tumors and the occurrence of a chronic inflammation endorse a critical role for tumor microenvironment on pancreatic carcinogenesis [4, 5]. In preclinical models, by releasing high amounts of growth factors such as granulocyte colony-stimulating factor (G-CSF) and granulocyte-macrophage colony-stimulating factor (GM-CSF), pancreatic tumor cells activate an abnormal myelopoiesis that promotes the recruitment of a heterogeneous population of myeloid cells characterized by a strong immunosuppressive activity [6, 7]. These cells are termed myeloid-derived suppressor cells (MDSCs) [8] and their accumulation, in the blood and at the tumor site, has been associated with advanced tumor stage and unfavorable prognosis in several human malignancies [9].

The main feature of MDSCs is the ability to switch off adaptive and innate immune responses [10]. Indeed, MDSCs are able to release both reactive oxygen species (ROS) and reactive nitrogen species (RNS), which inhibit T cells fitness, proliferation and migration within the tumor microenvironment; MDSCs deplete essential metabolites by activating key enzymes such as arginase-1 (ARG1) and indoleamine 2,3-dioxygenase 1 (IDO1), which are capable of reducing L-arginine and L-tryptophan availability, respectively. In addition, MDSCs induce T cell tolerance through the expression of inhibitory receptors such as the programmed death-ligand 1 (PD-L1) and the cytotoxic T-lymphocyte antigen 4 (CTLA-4) receptors, as well as they sustain the development of regulatory T cells (Treg) through the CD40 engagement in presence of inteleukin-10 (IL-10) and transforming growth factor beta (TGFβ) [11]. All these immunosuppressive mechanisms are the result of altered signaling pathways leading to induction of transcriptional factors such as the nuclear factor kappa-light-chain-enhancer of activated B cells (NF-κB) [12], the CCAAT-enhancer-binding proteins (c/EBP)-β [13] and members of the signal transducer and activator of transcription (STAT) family, like STAT3 [14]. Besides immune regulation, MDSCs favor tumor progression also by non-immune properties, promoting tumor angiogenesis and vasculogenesis as well as cancer cell stemness, aggressiveness and invasiveness [11].

In mice, MDSCs were classically identified as CD11b^+^Gr-1^+^ cells and divided into two main subgroups: polymorphonuclear (PMN)-MDSCs (CD11b^+^Ly6G^+^Ly6C^lo^ cells) and monocytic (M)-MDSCs (CD11b^+^Ly6C^+^Ly6G^−^ cells) [6]. In humans instead, three major MDSC subsets have been identified: PMN-MDSCs, M-MDSCs and “early-stage MDSCs” (e-MDSC) [15]. Since human MDSCs display surface markers shared with normal myeloid cell subsets (such as CD14, CD15, and CD33) and exhibit an intrinsic heterogeneity and plasticity, it is mandatory to integrate the phenotypical characterization with functional assays demonstrating their authentic immunosuppressive functions [15].

Here we applied standardized flow cytometry methods to distinguish and enumerate circulating MDSCs in both whole blood (WB) and frozen PBMCs obtained from three independent cohorts of PDAC patients; additionally, we analyzed the frequency of tumor-infiltrating MDSC and the immune composition in freshly isolated biopsies. Finally, we tested the immunosuppressive functions of circulating, purified MDSCs by evaluating their ability to control in vitro proliferation of activated T cells. Since only the M-MDSC subset showed robust inhibitory properties, we further exploited their transcriptomic profile with the aim at identifying novel biomarkers and specific molecular pathways.

## Material and methods

### Human samples collection

Peripheral blood samples were prospectively collected from three independent cohorts of patients with different stages of pancreatic ductal adenocarcinoma admitted at the Unit of General and Pancreatic Surgery of the Azienda Ospedaliera Universitaria Integrata of Verona before surgical resection or Healthy Donors (HD). Clinic-pathologic features of patients were reported in Tables 1 and 2 and included age, gender, tumor location and TNM stage. No subject had a prior history of cancer or was undergoing therapy at the time of sample collection. BM aspirates were subjected to lysis to remove red blood cells, with a hypotonic solution of ammonium chloride. Cells were plated (2 × 10^6^ cells/well) into a 24-well tissue culture plate (BD, Franklin Lakes, NJ, USA) in IMDM (Lonza, Visp, Switzerland) supplemented with with 10% FBS (Euroclone, Milano, Italy), 100 U/ml penicillin/streptomycin (Euroclone, Milano, Italy), β-mercaptoethanol (Sigma-Aldrich, Milan, Italy) and 10 mM HEPES (Euroclone, Milano, Italy) in presence of 40 ng/ml of G-CSF and GM-CSF (Miltenyi Biotec) for 4 days at 37 °C, 8% CO_2_, obtaining BM-MDSC as previously reported [16].

**Table 1:** Clinical characteristics of the study population

	Patients Cohort (*n* = 29)
Gender	
Male (%)	55.2
Female(%)	44.8
Age (Range)	66 (48–85)
Stage	
IIA-IIB (%)	48.3
III (%)	51.7
IV(%)	0.0
Tumor site	
Head	86.2
Body	10.3
Tail	3.5
Multi localized	0.0

The total number of cases, the male/female percentage, the mean age (years) with minimum and maximum value (range), stage and tumor localization of the analyzed cohort of PDAC patients

**Table 2:** Clinical characteristics of the study population

	Cohort_1 (*n* = 21)	Cohort_2 (*n* = 23)	Cohort_3 (*n* = 73)
Gender			
Male (%)	61.9	30.4	53.4
Female(%)	38.1	69.6	46.6
Age (Range)	67 (52–79)	67 (47–84)	65 (40–82)
Stage			
I-II (%)	/	/	28.7
III (%)	57.1	56.5	39.7
IV(%)	42.9	43.5	31.6
Tumor site			
Head	43.3	26.1	64.7
Body	29.0	47.8	19.1
Tail	19.0	17.4	6.8
Multi localized	8.7	8.7	9.4

The total number of cases, the male/female percentage, the mean age (years) with minimum and maximum value (range), stage and tumor localization of the three cohorts of PDAC patients. In each cohort, PDAC patients were compared to a cohort of age- and gender-matched healthy donors

### Human proliferation assay

PBMCs were isolated from leukocyte-enriched buffy coats from healthy volunteers (Transfusion Center, University and Hospital Trust of Verona, Verona, Italy) by Ficoll-Hypaque (GE Healthcare, Uppsala, Sweden) gradient centrifugation. PBMCs were then counted, frozen at − 80 °C and stored in liquid nitrogen. PBMCs were recovered, washed in IMDM medium (Lonza, Visp, Switzerland), supplemented with 10% FBS (Euroclone, Milano, Italy), 100 U/ml penicillin/streptomycin (Euroclone, Milano, Italy), β-mercaptoethanol (Sigma-Aldrich, Milan, Italy) and 10 mM HEPES (Euroclone, Milano, Italy), resuspended at a final concentration of 10^7^ cells/ml in PBS and stained with 1 μM as final working concentration of CellTrace Violet stock solution (Thermo Fisher Scientific, Waltham, MA, USA), followed by 5 min’ incubation at 37 °C, protected from light. Labelled “target” PBMCs were stimulated with coated 0.6 μg/ml anti-CD3 (clone OKT-3, eBioscience, Thermo Fisher Scientific, Waltham, MA, USA) and 5 μg/ml soluble anti-CD28 (clone CD28.2, eBioscience, Thermo Fisher Scientific, Waltham, MA, USA) for 4 days and co-cultured with “effectors” M-MDSCS (CD14^+^ cells) or PMN-MDSC (CD66b^+^ cells) cells at 0.5:1, 1:1, 3:1, 6:1 ratio (effector:target) in 384 flat bottom well plates (BD, Franklin Lakes, NJ, USA). Cell cultures were incubated at 37 °C and 8% CO_2_ in arginine and glutamine–Free-RPMI (Biochrom AG, Berlin, Germany), supplemented with 2 mM L-glutamine (Euroclone, Milano, Italy), 150 μM arginine (Sigma-Aldrich, St. Louis, MO, USA), 10% FBS (Superior, Merck, Darmstadt, Germany), 10 U/ml penicillin and streptomycin (Euroclone, Milano, Italy), and 0.1 mM HEPES (Euroclone, Milano, Italy). At the end of the culture, cells were stained with PE-Cy7 conjugated anti-CD3 (UCHT1, eBioscience, Thermo Fisher Scientific, Waltham, MA, USA), and CellTrace signal of gated lymphocytes was analyzed. TruCount™ tubes (BD, Franklin Lakes, NJ, USA) were used to determine the absolute cell number of CD3^+^ cells in the samples. Data were analyzed by FlowJo software (Tree Star, Inc. Ashland, OR, USA).

### Human cell preparation and flow cytometric analysis

Blood was collected into EDTA-treated tubes (BD Biosciences, NJ, USA) and processed fresh. For each donor, 450 μL of whole blood or 10^6^ frozen PBMCs were taken for MDSC characterization by flow cytometry. Sample tubes were washed in phosphate-buffered saline (PBS), incubated with Fc receptor (FcR) Blocking reagent (Miltenyi Biotec) for 10 min at 4 °C to saturate FcR and then stained with fluorochrome-conjugated antibodies (Additional file 1: Supplementary methods). For tumor-infiltrating leukocytes evaluation, tumor biopsies were minced and incubated for 2 h at 37 °C with shaking with an enzymatic cocktail. Normal tissues were detected by pathological analysis and isolated from patient biopsies. 5 × 10^5^ cells were washed with PBS supplemented with 2 Mm EDTA, incubated with FcR Blocking reagent (Miltenyi Biotec) for 10 min at 4 °C and then stained with fluorochrome-conjugated antibodies (Additional file 1: Supplementary methods).

### RNA isolation and gene expression

Total RNA was isolated using TRIzol reagent (Life technology, CA, USA) and RNA integrity assessed using Agilent-2100-Bioanalyzer (Agilent Technologies, CA, USA). RNA from human CD14^+^ cells was further purified with RNeasy MinElute Cleanup kit (Qiagen, Venlo, Netherlands) and cDNA was synthesized and amplified from total purified RNA with RETROscript® (Life technology, CA, USA). All the samples were hybridized to Affymetrix U133 PLUS 2.0 arrays and scanned with an Affymetrix GCS 3000 7G scanner.

### Statistical analysis

All statistical analysis was carried out using SigmaPlot (Systat Software) and R/Bioconductor. For statistical comparison of two groups, non-parametric Mann-Whitney Wilcoxon test was used. Data are shown as mean ± SD or mean ± SEM as indicated in the figures legends. Receiver operator characteristic (ROC) analysis was performed to determine the performance of MDSC percentage in distinguishing patients with metastatic carcinoma. The optimal cutoff threshold for MDSC percentage was obtained based on the maximization of the Youden’s statistics J = sensitivity+specificity+ 1 by using an R-based software as described [17]. Statistical analyses were performed using SPSS Statistics 22 (IBM Corporation, Somers, NY, USA), GraphPad Prism software program (version 6.0; GraphPad Software, San Diego, CA), and the statistical language R.

## Results

### The frequency of tumor-infiltrating T cells inversely correlates with the presence of PMNs and M-MDSCs

**Fig. 1:**
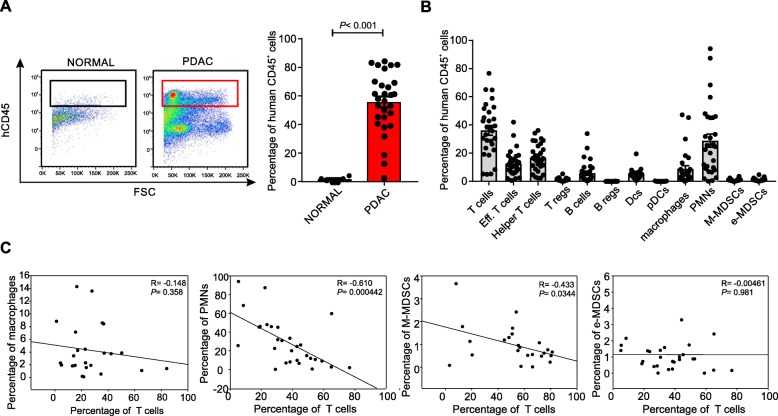
Immune characterization of PDAC tumor microenvironment. **a** Leukocytes infiltration (CD45^+^ cells) in normal pancreas (*n* = 5) and PDAC tissue (*n* = 29) biopsies. Statistical analysis was performed by ANOVA test. **b** Immune populations abundance (% of CD45^+^ cells) in PDAC tissues. **c** Correlation between tumor-infiltrating T cells with either macrophages, PMNs, M-MDSCs or e-MDSCs within PDAC tissues. Correlation analysis was performed by Spearman’s rank correlation

The immune composition of PDAC has been shown to have prognostic implications, with high number of CD8^+^ T lymphocytes associated with good outcome while accumulation of myeloid cells with poor prognosis [18, 19]. However, our knowledge on the immune heterogeneity of the PDAC microenvironment is still limited and need to be further investigated. To dissect this complexity, using a multicolour flow cytometry approach, we analysed infiltrating leukocytes isolated from 29 tumor samples from treatment-naïve PDAC patients (Table 1) and 5 normal pancreatic biopsies, obtained from tumor-free tissues of some patients. Among alive CD45^+^ cells, we focused on T lymphocytes (CD3^+^ cells), effector T lymphocytes (CD3^+^CD8^+^ cells), helper T lymphocytes (CD3^+^CD4^+^ cells), regulatory T lymphocytes (CD3^+^CD4^+^ CD25^+^FoxP3^+^ cells, Tregs), B lymphocytes (CD3^−^CD19^+^ cells), regulatory B cells (CD3^−^CD19^+^CD25^+^FoxP3^+^ cells, Bregs), myeloid-dendritic cells (CD11b^+^CD11c^+^HLA-DR^+^ cells, DCs), plasmacitoid DCs (CD11b^+^CD11c^−^CD123^+^, pDCs), macrophages (CD14^+^HLA-DR^+^CD68^+^CD206^+^ cells), granulocytes (PMNs, CD14^−^CD15^+^CD11b^+^ cells) as well as two MDSC subsets: e-MDSCs (Lin^−^HLA-DR^−^CD11b^+^CD33^+^ cells) and M-MDSCs (CD14^+^HLA-DR^−/lo^ cells) (Additional file 1: Figure S1). Notably, we found that PDAC tissues have a higher CD45^+^ cell infiltrate than their normal counterpart, likely reflecting the ability of the tumor or surrounding stroma to release soluble factors attracting immune cells [20, 21] (Fig. 1a). Among the CD45^+^ cells, we identified a high frequency of several myeloid cells, such as PMNs, MDSCs and macrophages (Fig. 1b) and several T cell subsets, supporting the current hypothesis that PDAC is not an immune “desert” [22, 23]. While we did not find expansion in Bregs (0.052 ± 0.012) and pDCs (0.073 ± 0.018), we observed a higher frequency of several myeloid cells, such as PMNs (28.89 ± 4.693), M-MDSCs (0.969 ± 0.167), e-MDSC (1.235 ± 0.198) and macrophages (8.832 ± 2.265) and Tregs (1.092 ± 0.196) (Fig. 1b), supporting the concept that PDAC is a tumor with an immune-hostile tumor microenvironment [24]. Indeed, a significant inverse correlation between T cell numbers with either PMN or M-MDSCs, but not with macrophages and e-MDSCs, could be detected (Fig. 1c), which is in line with recent reports. Of note, a significant inverse correlation between both PMNs and B cells, as well as between PMNs and different T cell subsets including effectors T cells, helper T cells and Tregs emerged (Additional file 1: Figure S2). Interestingly, we identified a significant direct linear correlation between T cells and Tregs as well as a trend between M-MDSCs and Tregs (Additional file 1: Figure S2). Collectively, these results suggest that accumulation of myeloid cells, such as MDSCs, in PDAC is detrimental for T cell infiltration.

### PDAC patients exhibit a significant increase in circulating MDSCs

**Fig. 2:**
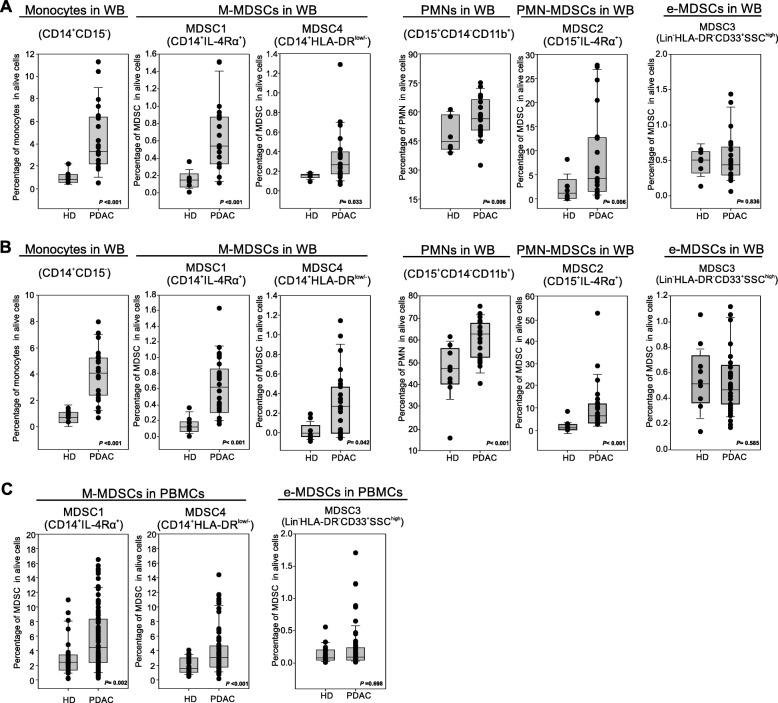
Blood-circulating MDSC enumeration in PDAC patients. **a-b** Flow cytometry analysis of circulating myeloid cells in whole blood of two independent cohorts of PDAC patients (**b** PDAC *n* = 21, HD = 8; **c** PDAC *n* = 23, HD = 9): monocytes (CD14^+^CD15^−^), MDSC1 (CD14^+^IL-4Rα^+^), MDSC4 (CD14^+^HLA-DR^low/−^), granulocytes (CD15^+^CD14^−^), MDSC2 (CD15^+^IL-4Rα^+^) and MDSC3 (LIN^−^HLA-DR^−^CD33^+^SSC^high^). **c** Flow cytometry analysis of circulating M-MDSCs (MDSC1, CD14^+^IL-4Rα^+^; MDSC4, CD14^+^HLA-DR^low/−^) and e-MDSCs (MDSC3, LIN^−^HLA-DR^−^CD33^+^SSC^high^) in PDAC patients (*n* = 73) compared to healthy donors (HD; *n* = 28). M-MDSC percentages were evaluated on frozen PBMCs, whereas e-MDSCs on the whole blood. Statistical analysis was performed by ANOVA test

Preclinical data suggest that PDAC effects on the immune system are not limited to local microenvironment but might cause systemic alterations, fuelling an “emergency” myelopoiesis that favours the accumulation of circulating MDSCs [13]. To assess systemic changes in PDAC patients, we evaluated the presence of different MDSC subsets in the peripheral blood, following recently published guidelines [25]. Three independent patient cohorts (Table 2) were enrolled to define the MDSC baseline at diagnosis: the first cohort comprised 21 PDAC patients (stage III-IV) and 8 age- and sex-matched healthy donors (HD) (Fig. 2a), the second cohort comprised 23 PDAC patients (stage III-IV) and 9 HDs (Fig. 2b); the last cohort consisted of 73 PDAC patients, including resectable (stage I-II, *n* = 21) and non-resectable (stage III-IV, *n* = 52) tumors and 28 HDs (Fig. 2c). MDSC analysis was performed using both fresh whole blood (WB) cells (Fig. 2a-b) and frozen peripheral blood mononuclear cells (PBMCs) (Fig. 2c). In fresh WB we analyzed the frequency of monocytes (CD14^+^CD15^−^CD11b^+^) and granulocytes (PMNs, CD15^+^CD14^−^CD11b^+^) as well as the presence of MDSC1 (CD14^+^IL-4Rα^+^), MDSC2 (CD15^+^IL-4Rα^+^), MDSC3 (Lin^−^HLA-DR^−^CD33^+^) and MDSC4 (CD14^+^HLA-DR^−/low^) subsets (Additional file 1: Figure S3). Among frozen PBMCs, we only discriminate monocytic (MDSC1 and MDSC4) and early-stage (MDSC3) MDSCs; the assessment of PMN-MDSCs is not accurate and probably even misleading, since granulocytes (including PMN-MDSCs) are typically lost during freezing/thawing procedure. We detected a significant increase in circulating M-MDSC subsets (MDSC1 and MDSC4) in PDAC patients compared to the control group in the three independent analyses (for CD14^+^IL-4Rα^+^ cells, median value 0.19% vs. 0.57%, *p* < 0.001 in the first cohort, 0.18% vs. 0.59%, *p* < 0.001 in the second cohort and 2.2% vs. 4.3%, *p* = 0.002 in the third cohort; for CD14^+^HLA-DR^−/low^ cells, median value 0.19% vs. 0.31%, *p* = 0.033 in the first cohort, 0.08% vs. 0.32%, *p* = 0.042 in the second cohort and 1.78% vs. 3.25%, *p* < 0.001 in the third cohort). Moreover PMN-MDSC subset (MDSC2) was significantly increased in PDAC patients: CD15^+^IL-4Rα^+^ cells, median value 1.53% vs. 4.89%, *p* = 0.006 in the first cohort, 1.89% vs. 6.78%, *p* < 0.001 in the second cohort. Interestingly, PDAC patients showed an increased frequency in WB of both monocytes and granulocytes compared to HDs: for monocytes, median value was 0.94% vs. 3.15%, *p* < 0.001 in the first cohort, 0.98% vs. 3.95%, *p* < 0.001 in the second cohort; for granulocytes, median value 44.82% vs. 56.23%, p = 0.006 in the first cohort, 47.89% vs. 62.45%, *p* < 0.001 in the second cohort. Finally, we did not observe any alteration in circulating e-MDSCs (MDSC3) between HDs and cancer patients in any of the analysed cohorts.

**Fig. 3:**
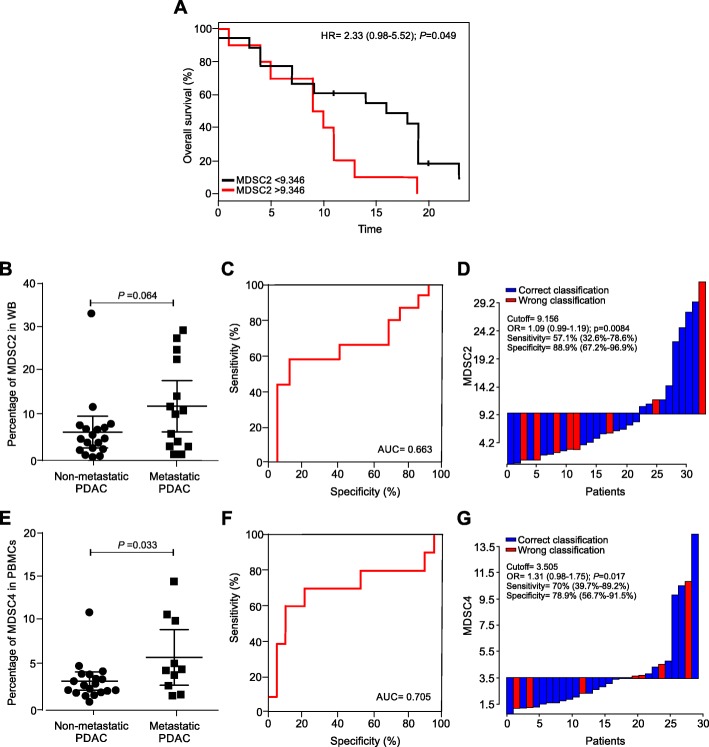
Prognostic potential predictive role of MDSCs in PDAC patients. **a** Kaplan–Meier curves for OS by significant MDSC2 cutoff frequency in fresh whole blood samples. **b** MDSC2 percentages in non-metastatic and metastatic PDAC patients. Mean and 95% confidence interval are plotted. Statistical analysis was performed by ANOVA test. **c** Receiver operator characteristic (ROC) curve for MDSC2 percentage in metastatic disease prediction. **d** Waterfall plot of optimal dichotomization; blue and red bars represent cases with correct or wrong classification, respectively. **e** MDSC4 percentage in non-recurrent and recurrent PDAC patients. Mean and 95% confidence interval are plotted. Statistical analysis was performed by ANOVA test. **f** Receiver operator characteristic (ROC) curve for MDSC4 percentage in metastatic disease prediction. **g** Waterfall plot of optimal dichotomization, blue and red bars represent cases with correct or wrong classification, respectively

To determine whether increased MDSC subsets could predict patient outcome, we analysed PDAC cohort 1 and 2 since these groups were homogeneously composed by poorly differentiated tumors (G3 and G4). The MDSC analysis was performed using the same sampling protocol based on fresh WB. We discovered that only MDSC2 frequency higher than optimal cutoff threshold (9.156%) was significantly associated with a shorter patient’s median overall survival (OS) (Fig. 3a) while the other analyzed MDSC subsets (MDSC1, MDSC3 and MDSC4) did not show any correlation with patients survival (Additional file 1: Figure S4). Moreover, a higher MDSC2 percentage is found in metastatic patients (*p* = 0.064, Fig. 3b) and significantly discriminated patients with metastatic disease with AUC value of 0.633 (*p* = 0.011, Fig. 3c) and an optimal cutoff threshold value of 9.156% (sensitivity of 57.1% (95% CI = 32.6–78.6%) and a specificity of 85.7% (95% CI = 65.4–95%)). In particular, 18 out of 24 (75%) patients with MDSC2 percentage lower than the identified cutoff did not present distant cancer dissemination whereas only 6 out of 24 (25%) patients presented clinically detectable metastases. Conversely, 8 out of 11 (73%) patients with MDSC2 percentage higher than the identified threshold presented metastases while 3 out of 11 (27%) patients had no metastasis (Fig. 3d). Starting from these premises, we evaluated the power of MDSCs from frozen PBMCs in discriminating patients with metastatic disease. Only MDSC4 frequency was able to significantly discriminate non-metastatic versus metastatic tumors (Fig. 3e) with AUC value of 0.705 (*p* = 0.017) (Fig. 3f) and a calculated threshold of 3.505% (sensitivity of 70% (95% CI = 39.7–89.2%) and a specificity of 78.9% (95% CI = 56.7–91.5%)). In fact, 15 out of 18 (83.3%) patients with MDSC4 percentage lower than the identified cutoff did not show metastases, whereas only 3 out of 18 (16.6%) patients present a metastatic disease; on the contrary, 7 out of 11 (63.6%) patients with MDSC4 percentage higher than the identified threshold had metastases, and 4 out of 11 (36.4%) patients did not show metastases (Fig. 3g). Collectively, these data suggest that MDSC percentage might be a valid prognostic biomarker of advanced disease in PDAC patients, even though the selection of the biomarker is strictly dependent on sampling: when the analysis is performed on cryopreserved PBMCs, MDSC4 levels could identify patients with metastatic disease, whereas MDSC2 frequency could stratify patients with metastases when the analysis is performed on fresh blood samples.

### Circulating monocytes from PDAC patients induce a stronger T-cell suppression compared to PMNs resembling effective M-MDSCs

**Fig. 4:**
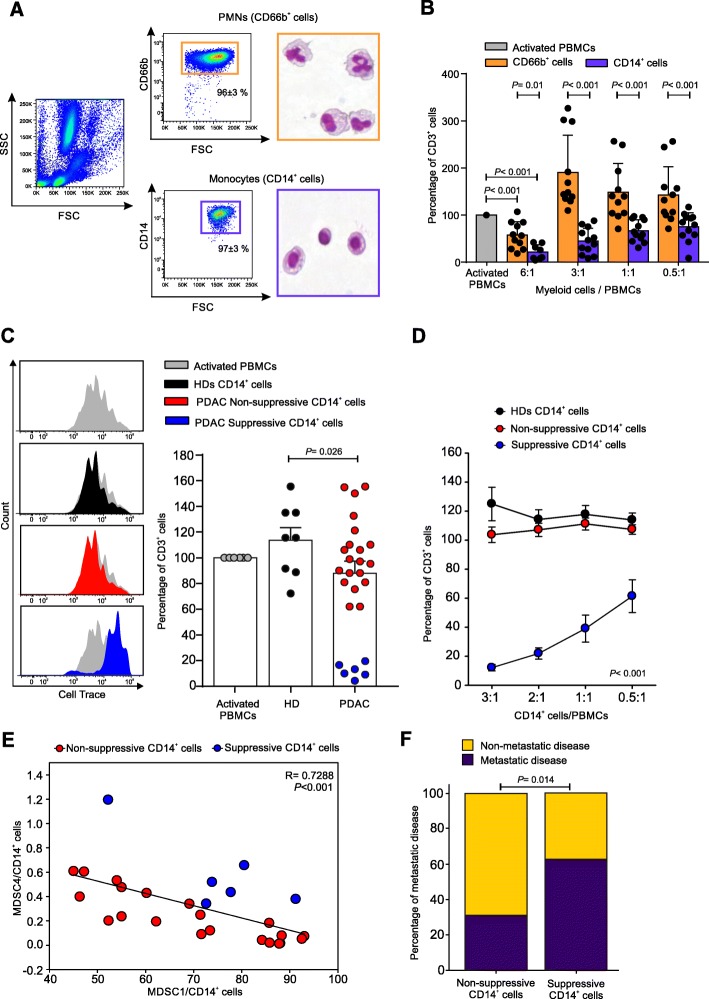
Circulating monocytes from PDAC patients are able to restrain T cell proliferation in vitro. **a** Freshly isolated PMNs (CD66b^+^ cells, orange box) and monocytes (CD14^+^ cells, blue box) from PDAC patients analysed by flow cytometry and haematoxylin-eosin staining. **b** Functional assay reflecting the different ability of PMNs and monocytes to affect T cells proliferation when co-cultured in vitro with CD3/CD28-activated-PBMCs at different ratios. All values are normalized on the activated PBMCs in the absence of myeloid cells (grey bar) and reported as percentage of Cell Trace^+^CD3^+^ cells. Statistical analysis was performed by ANOVA test. **c** Functional assay performed (at 1:3 ratio of PBMCs:CD14^+^ cells) on monocytes of PDAC patients (*n* = 26) compared to HDs (*n* = 8), reported as percentage of CD3^+^ proliferating cells (right panel) and graphed as proliferation peaks of Cell Trace^+^CD3^+^cells after the co-culture (left panel). Among all PDAC patients, “Suppressive CD14^+^ cells” (blue) and “Non-suppressive CD14^+^ cells” (red) were grouped based on the quantitative analysis of the in vitro immunosuppressive function. Statistical analysis was performed by ANOVA test. **d** Different ability of suppressive and non-suppressive monocytes to limit CD3^+^ T cell proliferation at different cell ratios. Statistical analysis was performed by ANOVA test. **e** Pearson correlation between MDSC4 and MDSC1 among CD14^+^ cells of PDAC patients. **f** Pro-metastatic potential of suppressive CD14^+^ cells. Statistical analysis was performed by Pearson Chi-Square test

We then evaluated in vitro the immunosuppressive properties of PMNs (isolated as CD66b^+^ cells) and monocytes (isolated as CD14^+^ cells) freshly purified from blood samples of the second PDAC patients cohort (*n* = 10) to confirm their MDSC-associated functional activity (cell purity was above 95% after cell isolation, Fig. 4a). Isolated cells were co-cultured in presence of activated, cell trace-labelled allogeneic PBMCs for 4 days. As reported in Fig. 4b, at the highest T cells:myeloid cells cell ratio (1:6) both myeloid cell subsets showed suppressive activity, whereas only monocytes were able to restrain T cell proliferation at lower cell ratio (i.e. at 1:1 ratio; *p* = 0.021 myeloid cells/PBMCs), in agreement with previous preclinical reports [6, 26]. Therefore, these data suggest that in PDAC patients, the per cell based suppressive capacity of neutrophils is lower than the one of monocytes, as it was already observed for other tumors [27].

We further validated the immunosuppressive activity of CD14^+^ cells purified from PDAC patients (*n* = 26) enrolled in the third cohort. Based on this analysis we stratified PDAC patients into two sub-groups: “suppressive PDAC” (*n* = 6, blue plots), whose monocytes were able to arrest T cells proliferation at different cell ratios (starting from 1:3 ratio of PBMCs:CD14^+^ cells), and non-suppressive/poorly suppressive PDAC (*n* = 20, red plots; hereafter referred as “non-suppressive PDAC”), in which CD14^+^ cells did not show any inhibitory properties similarly to HD-derived monocytes (*n* = 8, black plots) (Fig. 4c-d). We found that the presence of either suppressive or non-suppressive CD14^+^ cells did not correlate with any MDSC subsets: M-MDSC frequency (i.e. suppressive vs. non-suppressive: MDSC1/CD14^+^ cells median value 74.66 vs. 69.90, *p* = 0.53; MDSC4/CD14^+^ cells median value 5.78 vs. 4.09, *p* = 0.11) and the mean fluorescence intensity of IL-4Rα expressed on MDSC1 cells (i.e. suppressive vs. non-suppressive: median value 368.83 vs. 286.19, *p* = 0.44) did not correlate with immunosuppressive activity, as well. Moreover, we did not identify any clinical parameter able to discriminate the suppressive and not suppressive group of patients (i.e. those with immunosuppressive or not suppressive CD14^+^ cells). In fact, cell counts were not significantly different between the analyzed groups (suppressive vs. non-suppressive): WBE [10^9^/L] median value 5.89 vs. 6.08, *p* = 0.76; neutrophils [10^9^/L] median value 3.845 vs. 3.749, *p* = 0.86; monocytes [10^9^/L] median value 0.265 vs. 0.344, *p* = 0.16; lymphocytes [10^9^/L] median value 1.58 vs. 1.65, *p* = 0.58. Interestingly, we observed an inverse correlation comparing the frequency of MDSC1 and MDSC4 among monocytes (Fig. 4e), which allowed to distinguish the 80% of suppressive PDAC samples towards non-suppressive samples: suppressor monocytes showed simultaneously discrete amount of both MDSC-1 (MDSC1/CD14^+^ > 70%) and MDSC-4 (MDSC4/CD14^+^ > 0.2%), suggesting that both cell populations play a critical role in promoting a functional inhibition of T cell proliferation. Furthermore, the presence of suppressive CD14^+^ cells were able to significantly cluster metastatic versus non-metastatic patients: in fact, 11 out of 17 patients that exhibited suppressive monocytes (64.7%) presented a metastatic disease while this was found only in 8 out of 29 patients with non-suppressive monocytes (27.6%). Conversely, only 6 out of 17 patients presented a suppressive profile (35.3%) whereas 21 out of 29 patients with a non-suppressive profile (72.4%) exhibited a non-metastatic disease (Fig. 4f). Collectively, these data highlight that suppressive monocytes have a pro-metastatic potential.

### Immunosuppressive PDAC-derived CD14^+^ cells mainly activate a STAT3/arginase 1 axis

**Fig. 5:**
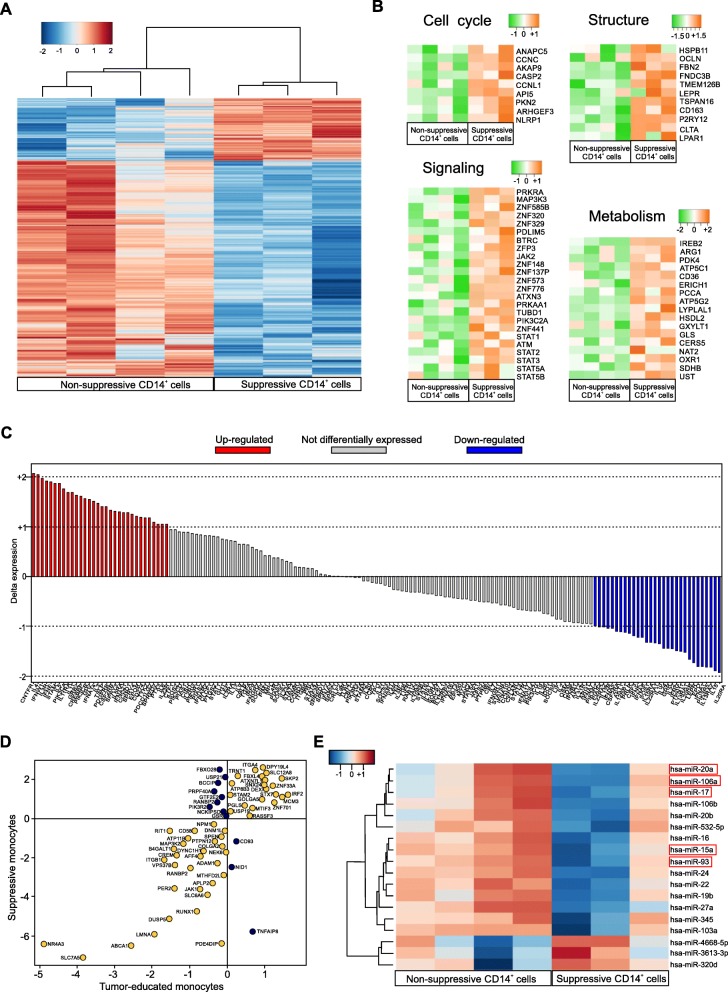
Gene profiling of suppressive CD14^+^ cells isolated from PDAC patient. **a** Supervised clustering of suppressive and not suppressive monocytes arrays using 1119 differentially expressed genes (FDR < 0.05 and absolute fold change > 2). **b** Clustering of cell cycle, structure, signaling and metabolism in suppressive- and not suppressive monocytes (absolute fold change > 2; FDR < 20%). **c** Difference in expression between suppressive monocytes isolated from PDAC patients and human BM-MDSCs samples for genes in JAK/STAT Signaling Pathway. **d** Dot plot of log fold change demonstrating common (yellow plots) or different (purple plots) gene expression modulation between differentially expressed signature of either tumor-educated or suppressive monocytes to related controls. **e** miRNAs-expression profile of suppressive and non-suppressive CD14^+^ cells isolated from PDAC patients using 19 differentially expressed miRNAs (FDR < 0.05 and absolute fold change > 2)

In order to define the molecular network relevant for CD14^+^ cell immunosuppression, we performed a genome-wide mRNA expression profiling on purified monocytes isolated from 3 suppressive and 4 non-suppressive PDAC patients. First of all, we compared the gene profiles of PDAC-derived monocytes against three independent public datasets of normal circulating CD14^+^ cells isolated from HDs (GSE60601, GSE64480 and GSE13899) demonstrating a specific cancer-related signature, as the hierarchical gene clustering revealed different patterns of expression between the two groups (Additional file 1: Figure S5A). Indeed, by using the gene set enrichment analysis (GSEA) of hallmarks of cancer, the differentially expressed genes were enriched in categories involved in: TNFα signaling via NF-κB, inflammatory response, IL6 JAK/STAT3 signaling and apoptosis categories (Additional file 1: Figure S5B). These results are in agreement with our recent findings [12] and indicate how cancer cells alter the normal monopoiesis favouring the development of CD14^+^ cells with cancer-related imprint. To elucidate further this cancer-driven reprogramming, we compared the gene profile of suppressive towards non suppressive PDAC monocytes by clustering genes according to their expression levels, demonstrating that immunosuppressive monocytes had a distinctive gene signature (Fig. 5a). The comparative analysis identified differences in the expression of genes involved in metabolism, cell cycle, signaling and structural components (Fig. 5b). Considering the structural constituents category, suppressive CD14^+^ cells showed a greater expression in *FBN2, TSPAN16, LEPR, CLTA* and *CD163* that are normally associated with classic monocytes. In particular, *CD163* expression is strongly regulated by IL-6 and IL-10 that are two of the main inflammatory mediators in PDAC patients’ sera [12, 21]. Moreover, the CD163 cleaved form (sCD163), released by monocytes/macrophages, was reported to inhibit T cell proliferation, underlying its potential involvement in immune evasion [28]. Suppressive monocytes showed also an altered cell cycle-associated gene signature, as well as a complex signaling-related gene enrichment. Among cell cycle cluster, we found the expression of *CASP2*, recently described as a regulating key of myeloid progenitor differentiation [29]; *AKAP9*, involved in c-AMP-dependent suppression on LPS-activated macrophages and *NLRP1*, described to impair T cell responses [30]. In the signaling category, we identified the expression of several zinc-finger protein-coding genes (*ZFP3, ZNF585B, ZNF320, ZNF329, ZNF148, ZNF137P, ZNF573, ZNF776* and *ZNF441*), as well as the different pattern of expression of *MAP 3 K3*, *PRKRA*, *JAK2* and different components of the STAT family (*STAT1, STAT2, STAT3, STAT5A, STAT5B* and *STAT6*) that have been already defined as MDSC-associated transcriptional factors [9]. In the metabolism group, we identified several genes potentially linked to immunosuppression [31]: fatty acid and lipoprotein metabolism-related genes, such as *CD36*, *LYPLA1* and *CERS5*; energy (ATP) metabolism-associated genes, such as *ATP51C, ATP5G2* and *SDHB*; glucose metabolism-associated genes, such as *PDK4* and *GXYLT1*, as well as hormone and water soluble vitamins metabolism-associated genes (i.e. *HSDL2* and *PCCA* respectively). Finally, we identified different genes involved in both amino acid metabolism, such as *ERICH1*, *GLS*, *CTSC* and *ARG1* and amino acid modifying enzymes, such as *NAT2*, *UST* and *OXR1*. To understand the depth of cancer-induced monocyte reprogramming towards MDSCs, we compared suppressive CD14^+^ cell gene profiles to gene signatures of human bone marrow (BM) derived MDSCs (BM-MDSCs, *n* = 8 independent donors) obtained by in vitro differentiation of BM cells in presence of a cytokine cocktail composed by G-CSF and GM-CSF, as previously reported [13] (Additional file 1: Figure S5C). Despite the phenotypic differences and the expected variances in their isolation and generation, BM-MDSCs and cancer patient immunosuppressive monocytes displayed a shared signature (not-differentially expressed genes, Fig. 5c) characterized by genes such as *PTGS2*, *SOCS2*, *TNF*, *IDO1, CD38* and *ARG1*, all related to immune regulation. Interestingly, they also shared expression levels of *AKT3*, *JAK1*, *JAK3*, *STAT1*, *STAT4*, *STAT5*, *STAT6* and *STAT3*, suggesting a common signaling network among these myeloid cells, as well as the same expression of *CFLAR,* which we recently reported as an important candidate for driving the acquisition of the immunosuppressive program in monocytes [12].

Notably, we identified a cluster of genes that are equally modulated in both suppressive monocytes and tumor-educated monocytes (recently described in [32]), suggesting a common tumor-dependent re-programming circuit (Fig. 5d). Among the most significant genes we identified *SKP2*, *IRF2* and *MCM3,* all related to tumor progression and metastases [33–35]. In agreement with these shared cues, 5 signaling pathways (MAPK, JAK-STAT, p53, VEGF and PI3K) that were not significantly different between immunosuppressive monocytes and tumor-educated monocytes, were observed; however, we found other signaling pathways uniquely upregulated in suppressive monocytes NF-κB, TGFβ, TNFα, Hypoxia, TRAIL and EGFR (Additional file 1: Figure S5D). Collectively, these data pinpoint suppressive monocytes as a peculiar subgroup of tumor-educated monocytes.

Finally, we integrated the transcriptome with a complete miRNAs profiling analysis of suppressive vs. non-suppressive PDAC CD14^+^ cells, using the same samples. The hierarchical clustering highlighted only 18 miRNAs that were differentially expressed between the two experimental groups (Fig. 5e). Surprisingly, among the down-regulated miRNAs in the suppressive CD14^+^ cells (*n* = 15), we identified *mir-17*, *mir-20a*, *mir-93*, *mir-106* and *mir-15a* that were reported to directly inhibit STAT3 [36, 37]. Indeed, these miRNAs are part of the 50 validated miRNAs able to bind the 3′-UTR region of STAT3 [37]. Therefore, these data allowed us to hypothesize that gain of suppressive function in MDSC could be partly dependent on the activation of a STAT3-dependent gene transcription.

**Fig. 6:**
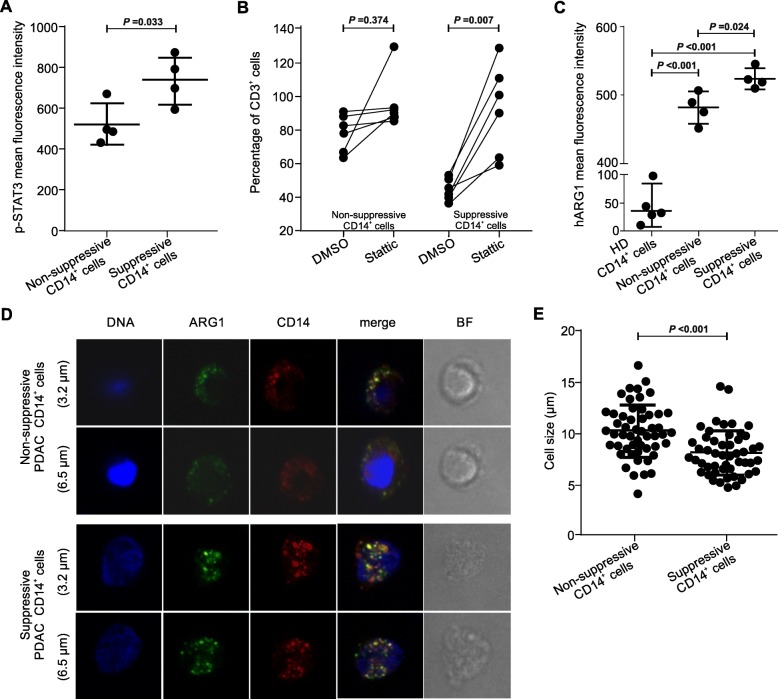
STAT3/ARG1 signaling is up-regulated in suppressive monocytes. **a** p-STAT3 detection in suppressive (*n* = 4) and non-suppressive (*n* = 4) PDAC patients’ monocytes was evaluated by flow cytometry. Statistical analysis was performed by ANOVA test. **b** Functional assay performed (at 1:3 ratio of PBMCs:CD14^+^ cells) on suppressive (*n* = 6) and non-suppressive (*n* = 6) monocytes of PDAC patients. CD14^+^ cells were treated with Stattic (5 μM) or DMSO for 30 min and, after the treatment, cells were washed three times and plated with T cells. Data are reported as percentage of CD3^+^ proliferating cells in three independent experiments. Statistical analysis was performed by ANOVA test. **c** ARG1 detection in suppressive (*n* = 4) and non-suppressive (*n* = 4) purified PDAC patients’ monocytes was evaluated by flow cytometry. As control, ARG1 expression in purified monocytes isolated from HDs is shown (*n* = 5). Statistical analysis was performed by ANOVA test. **d** Representative images of sorted, not suppressive or suppressive CD14^+^ cells obtained from patients with PDAC, stained for DNA (DAPI), ARG1 (green) and CD14 (red). BF = bright field. **e** Quantification of cell size by confocal microscopic analysis. Data shown 13 independent measures of each donors (*N* = 4). Statistical analysis was performed by ANOVA test

To prove the role of STAT3 among transcriptional factors driving MDSC function in PDAC, we first demonstrated an enhanced expression of the Tyr_705_-phosphorylated STAT3 (p-STAT3) in suppressive monocytes (Fig. 6a). Notably, treatment with Stattic, a specific small-molecule inhibitor of STAT3, significantly abrogated the suppressive activity of CD14^+^ cells, while it had no effects in non-suppressive monocytes, confirming the role of STAT3-driven program in MDSC-associated function (Fig. 6b). These results are consistent with data from Vasquez-Duddel et al. that demonstrated the therapeutic impact of Stattic on controlling MDSC function in head and neck squamous cell carcinoma [14]. Since p-STAT3 is able to bind different sites on the *ARG1* promoter to favor its transcription, we focused our next analyses on ARG1 expression. We measured ARG1 protein levels in both suppressive and non-suppressive CD14^+^ cells by flow cytometry and immunofluorescence (IF). We demonstrated that CD14^+^ARG1^+^ cells were significantly increased in cancer patients as compared to the HDs (Additional file 1: Figure S6A). However, they were not significantly different among suppressive vs. non-suppressive groups (median value 50.9 ± 3.25 vs. 48.6 ± 4.38; *p* = 0.76). We then measured the distribution and intensity of ARG1 by IF and we found a higher amount of ARG1 in suppressive than in non-suppressive monocytes (Fig. 6c). Additionally, confocal analysis showed a different pattern of ARG1 distribution, with the suppressive CD14^+^ cells being smaller and with diffused and less clustered ARG1-containing granules (Fig. 6d). By Z-stack analysis, suppressive CD14^+^ cells shared a significant smaller size (Fig. 6e), suggesting that immunosuppressive monocytes, that resemble M-MDSCs, may be clearly distinguished from monocytes present under steady-state hematopoiesis as small, ARG1^+^CD14^+^ cells.

## Discussion

Since the first identification of MDSCs at the end of 1970, both ontogeny and classification of those cells have been controversial [8]. In fact, while MDSCs share morphological features with conventional monocytes and granulocytes, they own the ability to dampen immune responses. Different reports have tried to identify unique MDSCs markers, but the newly identified subsets were rarely tested for inhibitory function, consequently missing the main characteristic to define those cells as MDSCs. Thus, following the recently published recommendations for MDSC characterization [15], we applied flow-cytometry technique to discriminate circulating MDSCs in PDAC patients and tested their ability to restrain T cell proliferation, in vitro. Interestingly, we unveiled an overall increased frequency of monocytes and PMNs in PDAC patients, probably as a consequence of tumor-induced inflammation. Among these cells, both M- and PMN-MDSC subsets were expanded in PDAC patients, both in the blood and tumor tissue, and overall, they were able to control T cell proliferation. Moreover, we demonstrated that CD14^+^ cells isolated from PDAC patients have a distinctive gene signature as compared to the monocytes isolated from HD suggesting that, during cancer progression, monocytes activate specific transcriptional programs that can be, in turn, used as potential biomarkers. Among patient-derived myeloid cells, we found CD14^+^ cells as the most potent myeloid subset to halt T cell proliferation, in line with previous preclinical data [26]. Notably, the functional assay allowed us to identify two distinct M-MDSC subgroups, i.e. suppressive and non-suppressive CD14^+^ cells. This cell discrimination was not connected with changes on the type and intensity of MDSC-associated markers but relied exclusively on functional suppressive ability mainly mediated by STAT3 activation. Therefore, functional tests cannot be considered as an adjunct to MDSC identification but, on the contrary, have a prioritizing role for definition of MDSC subsets. However, since standardizing functional tests among different laboratories might represent a challenging hurdle [38], we focused our subsequent efforts in identifying genes and molecular pathways that can represent clues to their immunosuppressive ability.

Further genetic analysis on M-MDSCs highlighted several other up-regulated targets linked to different molecular pathways, including the amino acid metabolism. In this gene-cluster we identified *ARG1*, which has been already associated to MDSC function in both tumor-bearing mice and cancer patients [39–41]. We recently reported that ARG1 has a hierarchical role in generating an immunosuppressive tumor microenvironment among the L-arginine metabolizing enzymes; indeed, myeloid cells expressing high levels of the inducible nitric oxide synthase (iNOS) enzyme (i.e. TNFα- and NO-producing dendritic cells) actively sustained anti-tumor T cells response and were counteracted by ARG1 activation in tumor-associated macrophages [42]. Differently, L-arginine deprivation, due to ARG1-expressing cells, strongly compromised both T cell proliferation and activation [43]. Moreover, ARG1-derived products, i.e. polyamines, could directly contribute to the generation of tolerogenic DCs through IDO1 phosphorylation, thus sustaining the immunosuppressive tumor microenvironment [44]. For the first time, our data demonstrated that ARG1 is expressed in human cancer-programmed monocytes, with the suppressive CD14^+^ monocytes expressing higher amount of the protein and presenting a unique pattern of staining, making them trackable from other circulating monocytes. Notably, circulating monocytes may acquire both ARG1 expression and MDSC-associated functions after tumor-derived exosomes uptake suggesting exosomes as tumor-derived cues to reprogram monocytes into immunosuppressive cells [45, 46]. Thus, ARG1-targeting approaches, using either ARG1 inhibitors or small molecules interfering with ARG1 transcription, such as AT38 or Stattic [14, 47–49], may offer a potential therapeutic options to the most aggressive form of PDAC. We think that targeting ARG1, with the new upcoming generation of engineered MDSC-specific nanoparticles, will be of great interest for many cancer types to unleash anti-tumor immunity. To this aim, we recently demonstrated the ability of newly designed lipid nanocapsules to vehicle, in vivo*,* chemotherapeutic agents exclusively to M-MDSCs, enhancing the therapeutic impact of immunotherapy [50].

Only in the recent past, PDAC was deeply interrogated at the genetic level, therefore revealing the presence of different molecular subtypes and, among them, an immune subgroup [51]. Moreover, the presence of a marked T cell-response against neo-antigen in PDAC microenvironment was associated to a better patients’ outcome and long term survival [22]. Finally, the recent efficacy of a combinatorial therapy, based on chemotherapy and immune checkpoint inhibition, in a mouse model of PDAC, open up to the development of several immunotherapeutic approaches that we hope could be successfully translated into the clinic, in the next years [52–54]. Of relevance are also recent results on TGFβ-targeted therapy showing synergism with anti-PD-L1-based treatment by dampening the tumor immunosuppressive microenvironment generated by peri-tumoral fibroblasts and favoring T cell trafficking to the tumor [55]. In many cancer patients, the lack of efficacy of several immunotherapeutic approaches, is related to the presence of an immunosuppressive network, mainly composed by MDSCs and macrophages that interferes with T cell trafficking and activation [24]. In line with these observations, our analysis of PDAC tumor-infiltrating leukocytes confirmed the negative association of these cells with T lymphocyte accumulation and activation. Therefore, we think that an effective immunotherapeutic approach, in PDAC patients, should combine the induction or transfer of tumor-specific T cells with the elimination of immunosuppressive cells. To this aim, several strategies might be explored to generate effective therapies, such as the use of low-dose chemotherapy, able to abrogate MDSC proliferation, accumulation and function [56, 57]; or, specific antibodies that limit myeloid migration into the tumor, such as monoclonal antibodies to CSF-1 receptor (CSF-1R), or antibodies to CCR2 and to CXCR4 [58], as well as the use of antibodies able to restrain tumor-induced inflammation (i.e. anti-IL-6 antibody).

## Conclusion

In conclusion, patient-derived M-MDSCs, identified as suppressive CD14^+^ cells, showing distinctive cytological features (a smaller size), functional properties (the ability to abrogate T cells) and gene signatures (i.e. activation of STAT3/ARG1 pathway) represent a peculiar branch within the complexity and heterogeneity of monocyte population found in tumors. We do not believe that MDSCs definition is an outdated concept, as recently postulated [59], but it rather defines a myeloid cell subset with unique properties, as we demonstrated in this work. It remains to be determined the tumor-derived factors that contribute to the development of this suppressive monocytes and we believe that single-cell technologies and fate mapping will help to reveal more information. In this regards, our data open a new insight in PDAC and in MDSC biology that may lead to more specific diagnosis and treatment for this lethal disease.

## Supplementary information

**Additional file 1.** Supplementary methods. **Figure S1.** Gating strategy to identify tumor-infiltrating leukocytes. **Figure S2.** Immune characterization of PDAC tumor microenvironment. **Figure S3.** Gating strategy to identify circulating MDSCs in fresh whole blood. **Figure S4.** Prognostic potential role of MDSCs in PDAC patients. **Figure S5.** Gene signature of CD14^+^ cells isolated from PDAC patients. **Figure S6.** Enumeration of circulating CD14^+^ARG1^+^ cells in PDAC patients.

## Data Availability

The datasets used and/or analyzed during the current study as well as reagents and samples are available from the senior authors on reasonable request and under material transfer agreement.

## Abbreviations

Ab: Antibody
APC: Allophycocyanin
APC-Cy7: Allophycocyanin cyanine 7
ARG1: Arginase-1
ATP: Adenosine triphosphate
AUC: Area under the roc curve
BM: Bone marrow
Bregs: Regulatory B cells
C/EBP: CCAAT-enhancer-binding proteins
c-AMP: Cyclic adenosine monophosphate
CCR: C-C chemokine receptor
CD: Cluster of differentiation
CFLAR: Caspase-8 and FADD like apoptosis regulator
CO_2_: Carbon dioxide
CSF: Colony stimulating factor
CTLA-4: Cytotoxic T-lymphocyte antigen 4
CXCR: C-X-C chemokine receptor
DC: Dendritic cells
EDTA: Ethylenediaminetetraacetic acid
FADD: Fas-associated protein with death domain
FBS: Fetal bovine serum
FcR: Fc receptor
FITC: Fluorescein isothiocyanate
FoxP3: Forkhead box P3
G-CSF: Granulocyte colony-stimulating factor
GM-CSF: Granulocyte-macrophage colony-stimulating factor
HD: Healthy donors
HEPES: 4-(2-hydroxyethyl)-1-piperazineethanesulfonic acid
IDO1: Indoleamine 2,3-dioxygenase 1
IF: Immunofluorescence
IL: Inteleukin
iNOS: Inducible nitric oxide synthase
JAK: Janus kinase
LPS: Lipopolysaccharide
M: Monocytic cells
MDSC: Myeloid-derived suppressor cells
MGG: May-Gruwald-Giemsa
mir: microRNA
NF-κB: Nuclear factor kappa-light-chain-enhancer of activated B cells
OS: Overall survival
PBMCs: Peripheral blood mononuclear cells
PDAC: Pancreatic ductal adenocarcinoma
pDC: Plasmacitoid DCs
PE: Phycoerythrin
PE-Cy7: Phycoerythrin cyanine 7
PMN: Polymorphonuclear cells
p-STAT3: Tyr_705_-phosphorylated STAT3
RNA: Ribonucleic acid
RNS: Reactive nitrogen species
ROC: Receiver operating characteristic
ROS: Reactive oxygen species
STAT: Signal transducer and activator of transcription
TGF-β: TGFtransforming growth factor beta
TNFα: Tumor-necrosis factor alfa
Treg: Regulatory T cells
UTR: Untranslated region
