# Supplementary material for: Immunosuppression by monocytic myeloid-derived suppressor cells in patients with pancreatic ductal carcinoma is orchestrated by STAT3
Source: J Immunother Cancer. 2019 Sep 18;7:255. doi: 10.1186/s40425-019-0734-6 (PMC6751612; doi:10.1186/s40425-019-0734-6)
Supplement: Supplementary file 1 — Additional file 1. Supplementary methods. Figure S1. Gating strategy to identify tumor-infiltrating leukocytes. Figure S2. Immune characterization of PDAC tumor microenvironment. Figure S3. Gating strategy to identify circulating MDSCs in fresh whole blood. Figure S4. Prognostic potential role of MDSCs in PDAC patients. Figure S5. Gene signature of CD14+ cells isolated from PDAC patients. Figure S6. Enumeration of circulating CD14+ARG1+ cells in PDAC patients. [file 40425_2019_734_MOESM1_ESM.pdf]

ADDITIONAL FILE

For additional files, please refer to:

SUPPLEMENTARY METHODS:

Immunomagnetic human MDSCs isolation.

Human $CD14^+$ or $CD66b^+$ cells were isolated by immunomagnetic sorting (using CD14 Microbeads and MACSxpress® Whole Blood Neutrophil Isolation Kit respectively, Miltenyi Biotec) according to manufacturer's instructions and their purity was evaluated by flow cytometry using mouse anti-human mAbs. For all separations, the positive fraction was obtained with a purity of ≥ 95%. BM-MDSCs were isolated trough 2 consecutive magnetic sortings: in the first round, BM-MDSCs were depleted of $CD3\varepsilon^+/CD19^+/CD56^+$ lymphocytes, with a cocktail of immunomagnetic beads obtained by combining anti-human CD3ε, CD19 and CD56 beads (Miltenyi Biotec). Subsequently, the $CD3\varepsilon^-/CD19^-/CD56^-$ fraction was enriched of $CD11b^+$ cells by positive selection with immunomagnetic anti-human CD11b beads (Miltenyi Biotec) as previously reported [1].

Flow Cytometry.

For whole blood and PMBCs cell labelling the following mAb were used: Fluorescein isothiocyanate (FITC) conjugated Lin cocktail [anti-CD14 (clone MϕP9), anti-CD16 (clone 3G8), anti-CD56 (clone NCAM16.2), anti-CD19 (clone SJ25C1), anti-CD3 (clone SK7), anti-CD20 (clone L27), BD Biosciences], Phycoerythrin (PE) conjugated anti-CD124 (clone FAB230P, R&D), Peridinin-chlorophyll proteins/ Cyanine 5.5 (PERCP/Cy5.5) conjugated anti-HLA-DR (clone L243, eBioscience, Thermo Fisher Scientific), Phycoerythrin Cyanine 7 (PE-Cy7) conjugated anti-CD11b (clone ICRF44, BD Biosciences), Allophycocyanin (APC) conjugated anti-CD33 (clone WM53, BD Biosciences) Allophycocyanin-Cyanine 7 (APC-Cy7) conjugated anti-CD14 (clone M5E2, BD Biosciences) and Aqua conjugated (Invitrogen,Thermo Fisher Scientific) Live/Dead fixable staining for 30 minutes at 4°C.

For p-STAT3 detection, $5x10^5$ frozen PBMCs were thawed and kept 1h at 37°C. Sample tubes were then washed in PBS and incubated with FcR Blocking reagent (Miltenyi Biotec) for 10 minutes at 4°C to saturate FcR. Cells were stained with Abs specific for surface markers (CD14, clone M5E2; and CD3, clone SK7) and Live/Dead reagent. Samples were than fixed with 2% of paraformaldehyde for 10 minutes at 37°C, and permeabilized using 250µl of methanol 90% for 30 minutes at 4°C. The intracellular staining was then performed using PE-conjugated anti-pSTAT3 (clone Y705, Cell Signaling Technologies) at 1:50 in PBS for 1h at room temperature.

For Arg1 detection, $1x10^6$ of frozen purified $CD14^+$ cells were thawed in FBS and incubated with FcR Blocking reagent (Miltenyi Biotec) for 10 minutes at 4°C to saturate FcR. Cells were stained with the following Ab mix: anti-CD14 (clone MφP9) plus anti-CD3 (clone SK7). For the intracellular staining, cells were fixed and permeabilized using the BD Cytofix/Cytoperm kit according to the manufacturer's instructions. The monoclonal Alexa-Fluor647-conjugated; anti-Arginase-1 (AF647, clone 1.10, hybridoma homemade) was added for 1h. For tumor-infiltrating leukocytes evaluation the following mAbs were used for cell labelling: FITC-conjugated Lin1 cocktail [anti-CD14 (clone MφP9), anti-CD16 (clone 3G8), anti-CD56 (clone NCAM16.2), anti-CD19 (clone SJ25C1), anti-CD3 (clone SK7), anti-CD20 (clone L27), BD Biosciences], PE-conjugated anti-CD25 (clone BC96, Thermo Fisher Scientific), PE-conjugated anti-CD14 (clone M5E2, BioLegend), PE-conjugated anti-CD123 (clone 6H6, eBioscience, Thermo Fisher Scientific), PERCP/Cy5.5-conjugated anti-HLA-DR

(clone L243, eBioscience, Thermo Fisher Scientific), PERCP/Cy5.5-conjugated anti-CD3 (clone UCHT1, BD Biosciences), PE-Cy7-conjugated anti-CD11c (clone 3.9, eBioscience, Thermo Fisher Scientific), PE-Cy7-conjugated anti-CD19 (clone SJ25C1, BD Biosciences), PE-Cy7-conjugated anti-CD11b (clone ICRF44, BD Biosciences), APC-conjugated anti-CD33 (clone WM53, BD Biosciences), APC-conjugated anti-CD8 (clone RPA-T8, BD Biosciences), APC-conjugated anti-CD206 (clone 19.2, BD Biosciences), APC-Cy7-conjugated anti-CD4 (clone SK3, eBioscience,Thermo Fisher Scientific), APC-Cy7-conjugated anti-CD11b (clone ICRF44, BD Biosciences), V450-conjugated anti-CD15 (clone HI98, BD Biosciences), V450-conjugated anti-CD45 (clone HI30, BD Biosciences), eFluor® 780-conjugated (eBioscience,Thermo Fisher Scientific) and Aqua conjugated (Invitrogen,Thermo Fisher Scientific) Live/dead fixable staining for 30 minutes at 4°C. For intracellular markers evaluation (FoxP3 and CD68) cells were fixed and permeabilized with FoxP3 / Transcription Factor Staining Buffer Set according to manufacturer's instructions (eBioscience, Thermo Fisher Scientific). FITC-conjugated anti-FoxP3 (clone 259D/C7, BD Biosciences) or FITC-conjugated anti-CD68 (clone Y1/82A, BD Biosciences) were added for 30 minutes at 4°C. Samples were acquired with a FACSCanto II (BD, Franklin Lakes, NJ, USA) and analyzed with FlowJo software (Treestar Inc.).

Cytospin preparation and may-Gruwald-Giemsa (MGG) staining.

Sorted cells were centrifuged (Shandon Cytospin 3 centrifuge) on microscope slides, and cytospins were stained and analysed as previously reported [1].

Analysis of gene expression data

In-depth analyses and clustering of data were conducted in R/Bioconductor. After data acquisition, normalization was performed using quantile procedure and genes that were consistently absent or below the noise level were excluded from analysis. To identify genes with statistically significant differences between the comparison of the group of interest, we performed the empirical Bayes moderated t-test as implemented in the LIMMA R-package [2] with a p value cut-off of 0.05 and the Benjamin and Hochberg false discovery rate, as multiple testing correction. Hierarchical clustering was performed on both genes and individual samples, with Euclidian distance as a measure of similarity to group genes and samples with similar expression patterns. Data points were arranged in a hierarchy and were displayed in a phylogenetic tree of clusters of genes in a hierarchically ordered relationship. Branch lengths represent the degree of similarity between sets and gene expression profiles that were similar across the experimental samples were clustered together. The Affymetrix platform for miRNA expression analysis (GeneChip miRNA 3.0 array), based on miRBase 17 (http://www.mirbase.org/), was used to obtain miRNA profiles. Normalization and statistical analysis were performed in R/Bioconductor using gcrma, affy and limma packages. Briefly, raw data probes were normalized using rma algorithm. The normalized expression values were log2 transform and use as input of Limma to generate lists of differentially expressed miRNA. Finally, PROGENy (Pathway RespOnsive GENes) algorithm was applied to evaluate cancer-associated signalling pathways, using signatures of consensus genes.

Confocal microscopic analysis.

FACS sorted $CD14^+$ cells were let adhere on 14-mm round Menzel-Glaser glass for 2h then fixed with 4% paraformaldehyde for 10 minutes at room temperature. After extensive wash with PBS,

the cells were incubated for 1 hour at room temperature with PBS containing FcR blocking reagent (Miltenyi) diluted 1:25. Cells were then stained with anti-CD14 FITC (clone TUK4, Miltenyi, diluted to 1:20), anti-ARG1 AF647 (hybridoma clone 1.10, homemade and directly conjugated with Alexa Fluor-647, diluted 1:1000) in PBS for 2h at room temperature, in the dark. Slides were then washed with PBS 0.05% Tween- 20 and cells were then stained with 4′,6-diamidino-2-phenylindole (DAPI, Sigma-Aldrich) diluted 1:500 in PBS for 10 minutes at room temperature, in the dark. After extensive washes with PBS, coverslips were mounted with ProLong Gold antifade Mounting media (ThermoFisher Scientific) in Superfrost Plus adhesion microscope slides (ThemoFisher Scientific) and acquired by confocal microscopy (TCS SP5, Leica Microsystems CMS GmbH, Wetzlar, Germany). Cells were located and positioned using bright field illumination (BF). Fluorescence images were captured sequentially, using a 405-nm laser line for DAPI, a 488-nm laser line for FITC and 633-nm laser line for Alexa Fluor 647. Images were analyzed by LAS AF Lite 2.0.2 (Leica Microsystems CMS GmbH) and NIH-Image J programs (Bethesda, USA). Images (512x512 pixels in TCS SP5 system) were acquired with an oil immersion objective (63× in TCS SP5 system; NA = 1.35). 10 different regions of each coverslip were taken randomly. Exposure times of each channel were kept constant over the whole series after calibrating on a bright representative sample to avoid saturated pixels.

SUPPLEMENTARY REFERENCES

1. Solito, S., et al., *A human promyelocytic-like population is responsible for the immune suppression mediated by myeloid-derived suppressor cells.* Blood, 2011. **118**(8): p. 2254-65.
2. Smyth, G.K., Y.H. Yang, and T. Speed, *Statistical issues in cDNA microarray data analysis.* Methods Mol Biol, 2003. **224**: p. 111-36.

SUPPLEMENTARY FIGURES

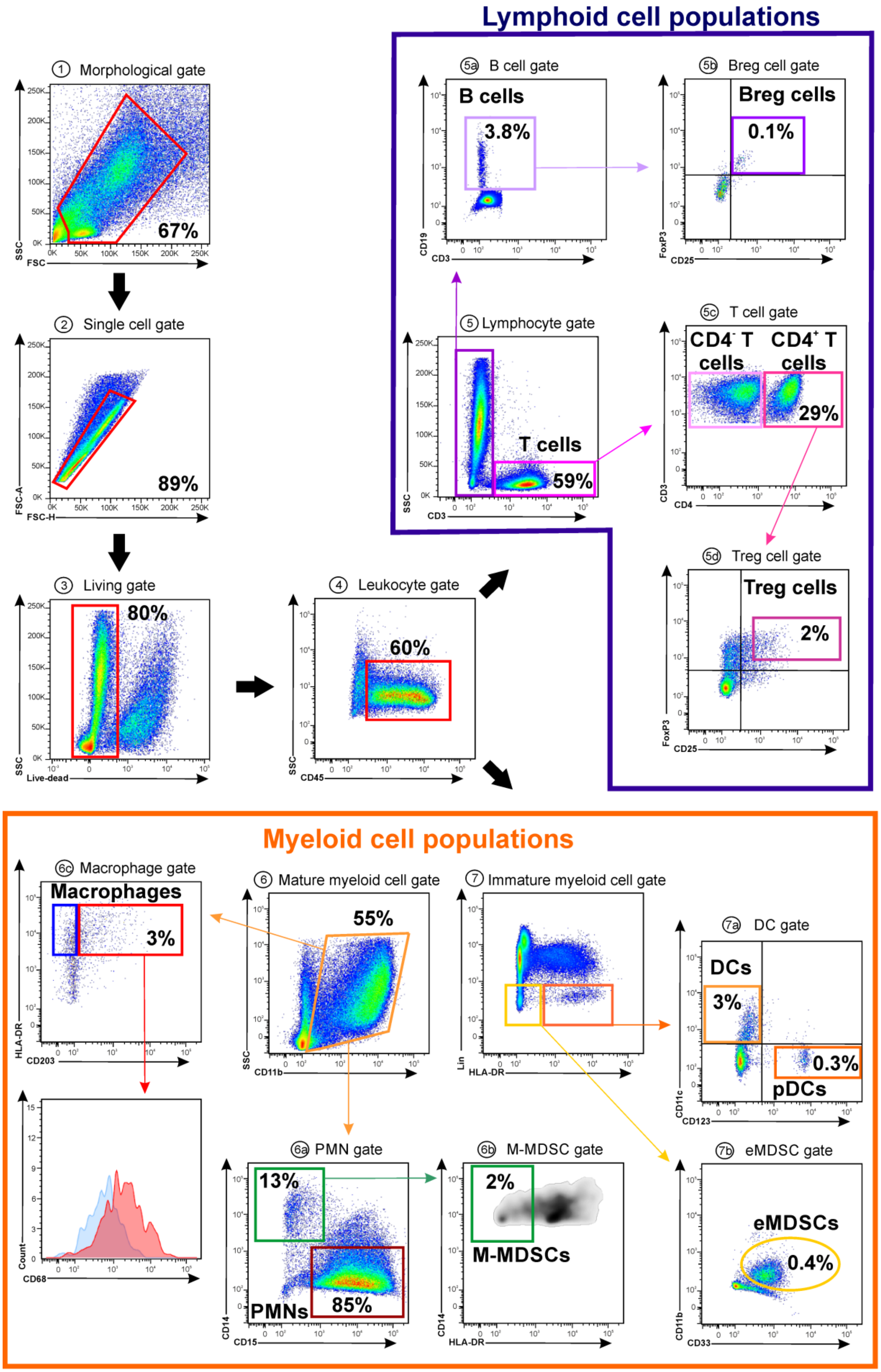

Supplemetary Figure_1

Figure S1. Gating strategy to identify tumor-infiltrating leukocytes. Pancreatic tissues were minced and incubated for 2 hours at 37°C shaking with an enzymatic cocktail. PDAC-infiltrating cells were stained and analysed by flow cytometry using a gate strategy based on consecutive gates: 1) morphological gate, 2) single cells gate, 3) living gate (Live/Dead$^-$ cells) and 4) leukocytes gate ($CD45^+$ cells). For the detection of lymphoid populations, $CD45^+$ cells were analyzed by a lymphocyte gate (5), that identified T cells ($CD3^+$ cells) and no-T cells as $CD3^-$ cells in which we detected B cells ($CD19^+$ cells, 5a) and Breg cells ($CD19^+CD25^+FoxP3^+$ cells, 5b). Among T cells we detected effector T cells ($CD3^+CD4^-$ cells, 5c) and helper T cells ($CD3^+CD4^+$ cells, 5c). Finally, among helper T cells we detected Treg cells ($CD3^+CD4^+CD25^{high}FoxP3^+$ cells, 5d). For the detection of myeloid populations, $CD45^+$ cells were analyzed by a mature myeloid cell gate (6), that identified $CD11b^+$ cells. These cells were analyzed as $CD14^-CD15^+$ cells, that identified PMNs (6a); $CD14^+CD15^-HLA\text{-}DR^-$ cells, that identified M-MDSCs (6b); and $CD11b^+CD203^+CD68^+$ cells, that identified macrophages (6c). Leukocytes were also analyzed by an immature myeloid cell gate (7), that identified three different cell subsets: $Lin^-HLA\text{-}DR^-CD33^+$ cells, that identified eMDSCs (7b); $Lin^-HLA\text{-}DR^+CD11c^+CD123^-$ cells, that identified DCs (7a); and $Lin^-HLA\text{-}DR^+CD11c^-CD123^+$ cells, that identified pDCs (7b). Shown percentages refer to a representative sample.

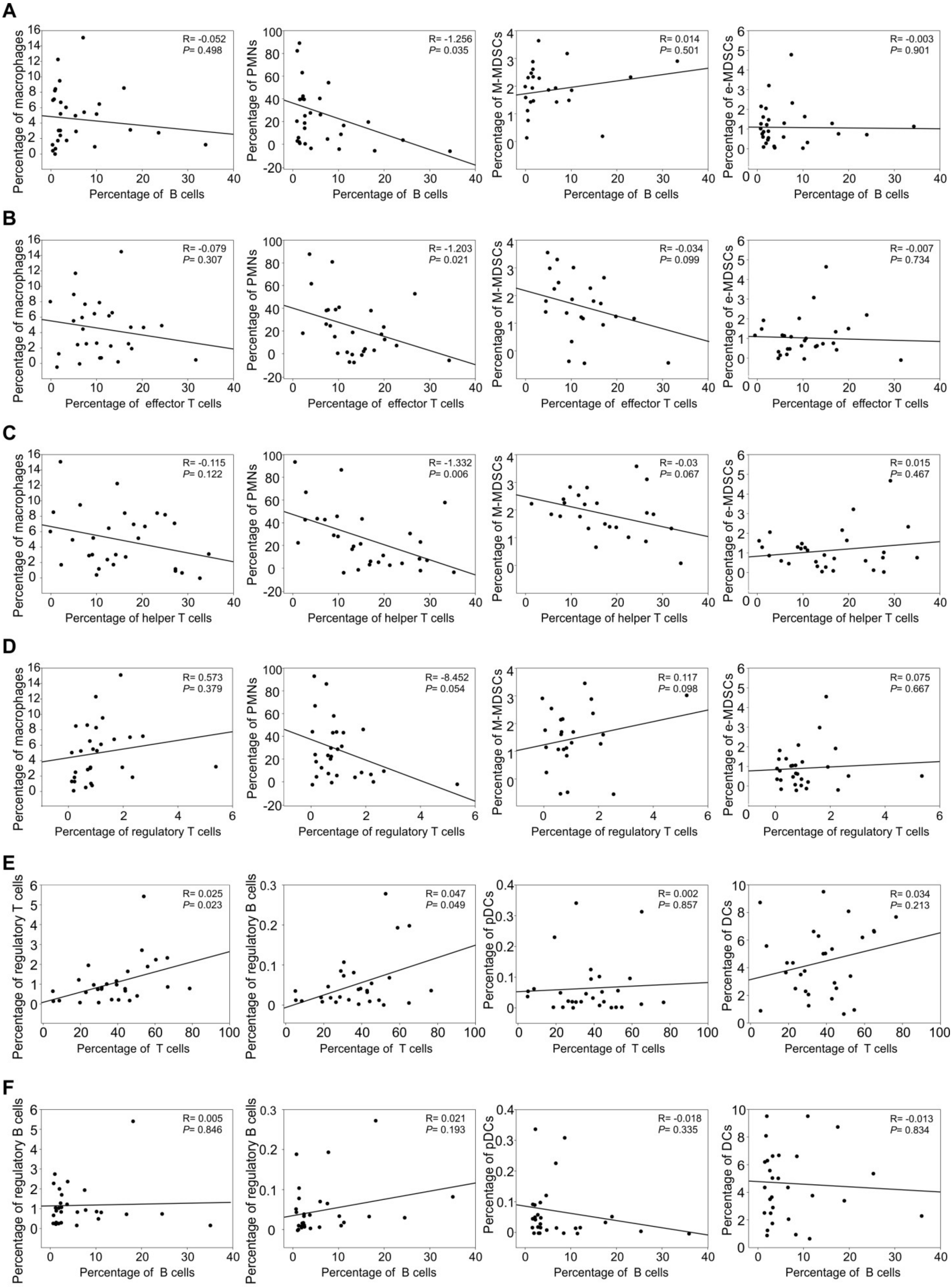

Supplemetary Figure_2

Figure S2. Immune characterization of PDAC tumor microenvironment.

(A) Spearman's rank correlation between tumor-infiltrating B cells with either macrophages, PMNs, M-MDSCs or e-MDSCs within PDAC tissues. (B) Spearman's rank correlation between tumor-infiltrating effectors T cells with either macrophages, PMNs, M-MDSCs or e-MDSCs within PDAC tissues. (C) Spearman's rank correlation between tumor-infiltrating helper T cells with either macrophages, PMNs, M-MDSCs or e-MDSCs within PDAC tissues. (D) Spearman's rank correlation between tumor-infiltrating regulatory T cells with either macrophages, PMNs, M-MDSCs or e-MDSCs within PDAC tissues. (E) Spearman's rank correlation between tumor-infiltrating T cells with either regulatory T cells, regulatory B cells, pDCs or DCs within PDAC tissues. (F) Spearman's rank correlation between tumor-infiltrating B cells with either regulatory T cells, regulatory B cells, pDCs or DCs within PDAC tissues.

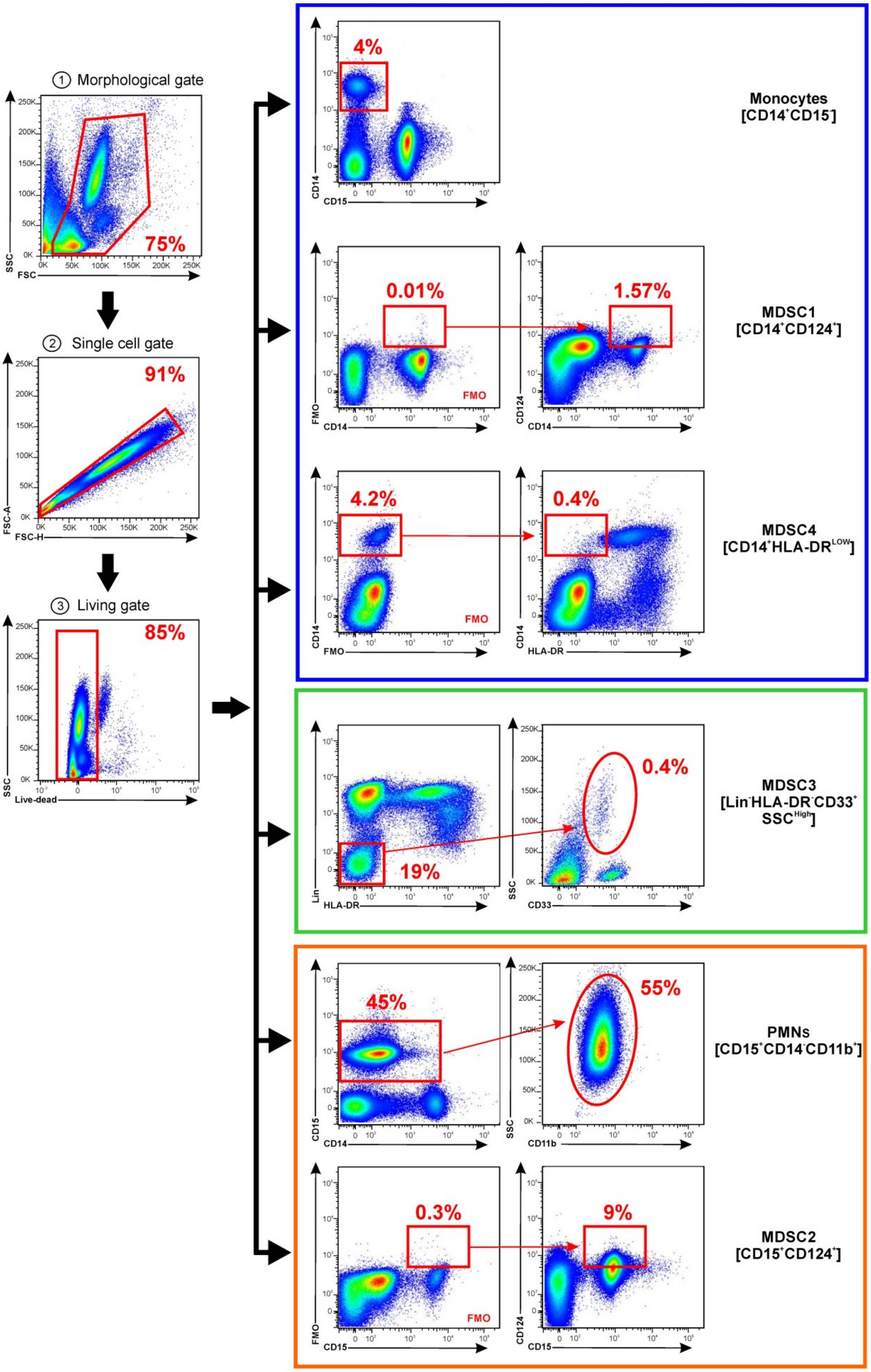

Supplemetary Figure_3

Figure S3. Gating strategy to identify circulating MDSCs in fresh whole blood. Circulating leukocytes were stained and analysed by flow cytometry using a gating strategy based on the following consecutive gates: 1) morphological gate, 2) single cells gate and 3) living gate (Live/Dead). These gating strategy allows to discriminate monocytes and M-MDSCs (blue square), e-MDSCs (green square) and PMNs and PMN-MDSCs (orange square). Shown percentages refer to a representative sample.

A

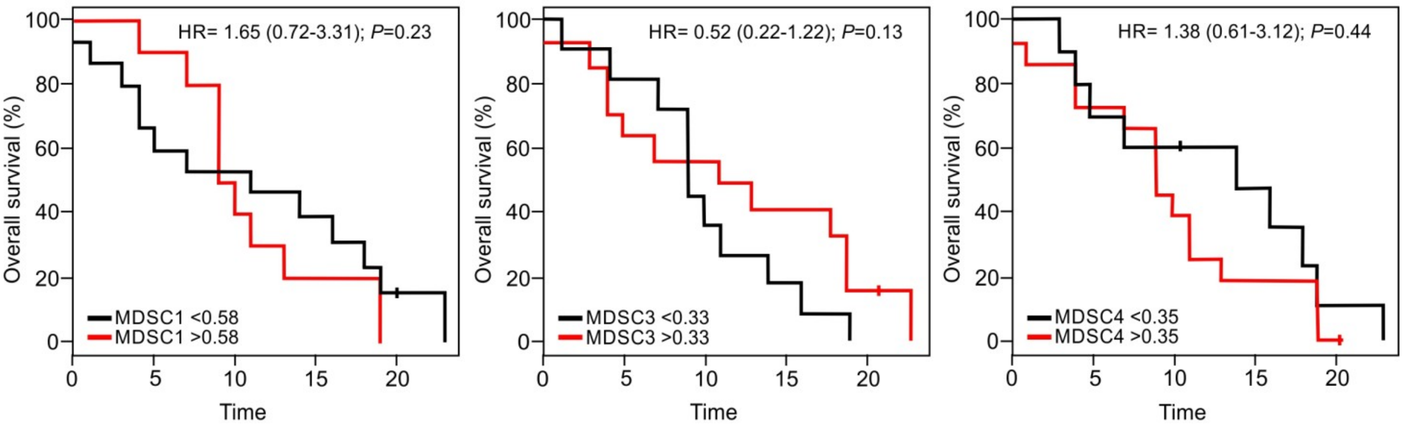

268

Supplemetary Figure_4

Figure S4. Prognostic potential role of MDSCs in PDAC patients.

(A) Kaplan–Meier curves for OS by significant cutoff frequency of MDSC1 (0.58), MDSC3 (0.33) and MDSC4 (0.35) in fresh whole blood samples.

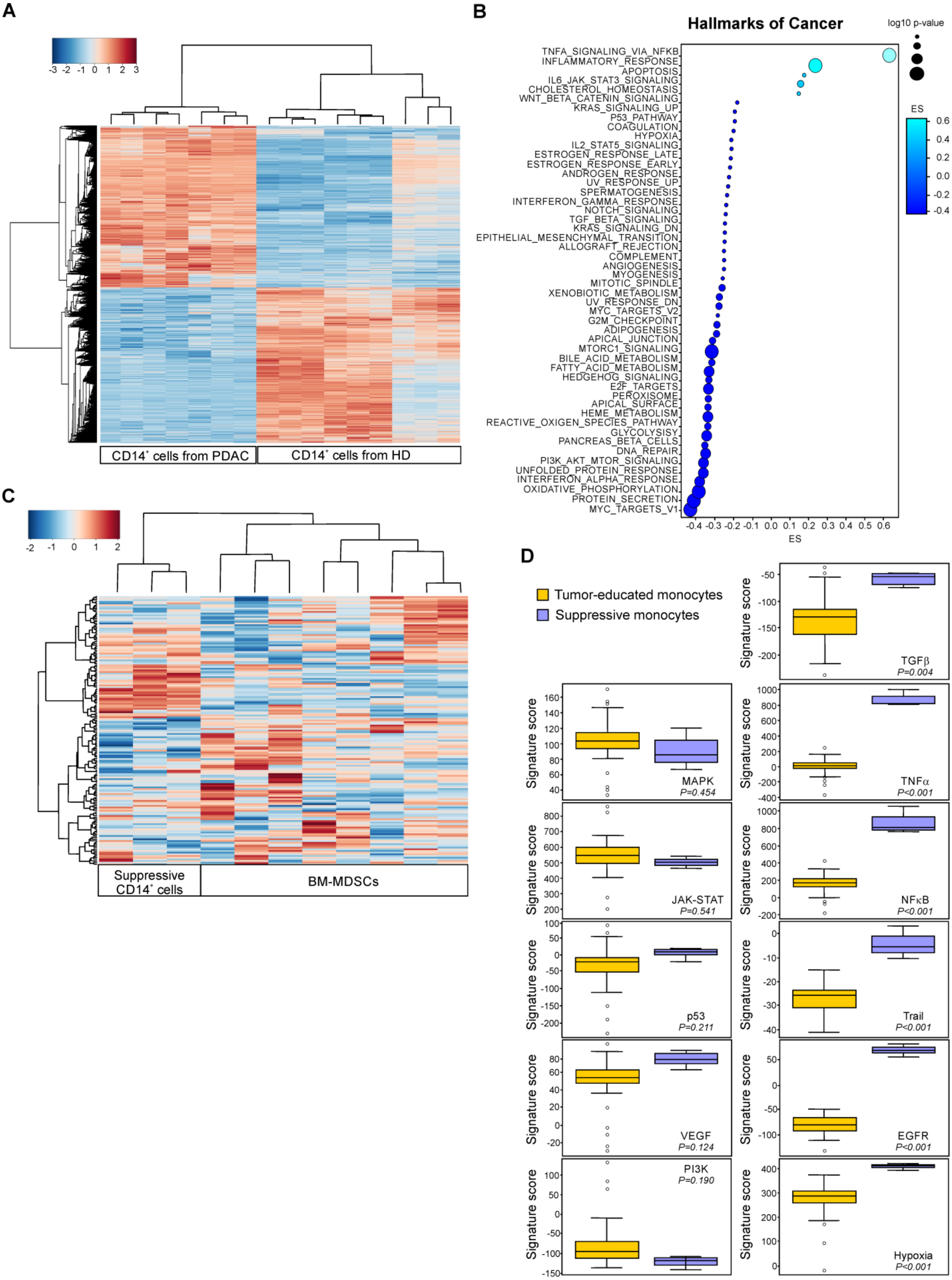

Supplemetary Figure_5

Figure S5. Gene signature of CD14$^+$ cells isolated from PDAC patients.

(A) Supervised clustering of CD14$^+$ cells from PDAC using 1,500 differentially expressed genes (FDR<0.05 and absolute fold change >2) with public datasets of normal circulating CD14$^+$ cells isolated from HDs (GSE60601, GSE64480 and GSE13899). (B) Enrichment score (ES) and p-value of the 50 Hallmarks of cancer associated to monocytes from PDAC patients. (C) Supervised clustering of suppressive CD14$^+$ cells from PDAC patients and BM-MDSCs (n=8) using 1,322 differentially expressed genes (FDR<0.05 and absolute fold change >2). (D) Box plots of common (left panels) and differentially expressed (right panels) cancer-related signaling pathways between tumor educated monocytes from public datasets (GSE117970) and suppressive CD14$^+$ cells of PDAC patients using PROGENy software.

A

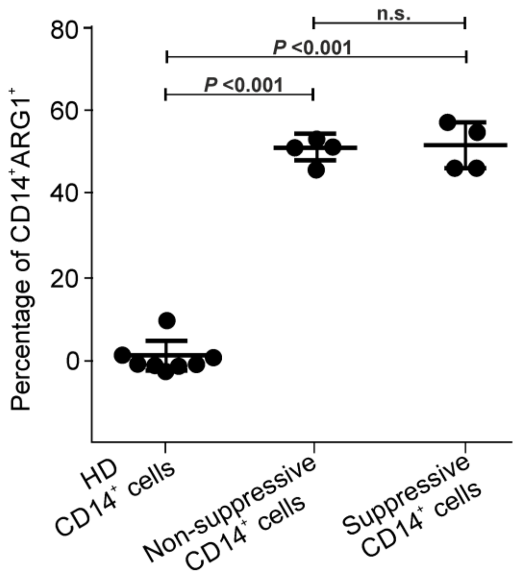

Supplemetary Figure_6

Figure S6. Enumeration of circulating CD14$^+$ARG1$^+$ cells in PDAC patients.

(A) Flow cytometry analysis of CD14$^+$ARG1$^+$ cells in sorted circulating monocytes (CD14$^+$ cells) of PDAC (n=8) patients and HDs (n=8). Statistical analysis was performed by ANOVA test.
